# Reconfigurable Ring Filter with Controllable Frequency Response

**DOI:** 10.1155/2014/671369

**Published:** 2014-07-08

**Authors:** Norfishah Ab Wahab, Mohd Khairul Mohd Salleh, Zuhani Ismail Khan, Nur Emileen Abd Rashid

**Affiliations:** Microwave Technology Centre, Faculty of Electrical Engineering, Universiti Teknologi MARA (UiTM), 40150 Shah Alam, Selangor, Malaysia

## Abstract

Reconfigurable ring filter based on single-side-access ring topology is presented. Using capacitive tuning elements, the electrical length of the ring can be manipulated to shift the nominal center frequency to a desired position. A synthesis is developed to determine the values of the capacitive elements. To show the advantage of the synthesis, it is applied to the reconfigurable filter design using RF lumped capacitors. The concept is further explored by introducing varactor-diodes to continuously tune the center frequency of the ring filter. For demonstration, two prototypes of reconfigurable ring filters are realized using microstrip technology, simulated, and measured to validate the proposed concept. The reconfigured filter using lumped elements is successfully reconfigured from 2 GHz to 984.4 MHz and miniaturized by 71% compared to the filter directly designed at the same reconfigured frequency, while, for the filter using varactor-diodes, the frequency is chosen from 1.10 GHz to 1.38 GHz spreading over 280 MHz frequency range. Both designs are found to be compact with acceptable insertion loss and high selectivity.

## 1. Introduction

Modern and integrated communication systems are pushing for compact, low cost with flexible design for front-end electronic components. This led to the evolution of bandpass filters' construction with various types of topologies and technologies. Amongst these, miniaturized and tunable bandpass filters have the potential for further improvement to fit the advancement of technology with rigid specifications in communication systems. Numerous techniques have been explored and amongst these are high performance reconfigurable filters with simple topology, fast tuning speed, sharp rejection skirt for high selectivity, and compact size that received great interests [1–4]. Well-established tuning methods made use of devices such as RF microelectrical mechanical systems (MEMS) and ferroelectric-based and magneto-electric devices using Yttrium Iron Garnet films (YIG) and reactive elements were reported in [1–6]. Undeniably, various resonator shapes or topologies can be designed easily, but it is not a simple task to couple these elements on a microstrip resonator. The tuning elements must be carefully arranged and coupled to the resonator to minimize the filter size and losses. Furthermore, most of the work did not elaborate or neglect the synthesis part which is important for flexible design [7–11].

Therefore, this paper proposed a simple topology using single-side-access ring resonator as a base cell. The advantage of this single-side-access ring resonator is its compactness and simple configuration with minimum number of controlling parameters besides high selectivity characteristic. The ring is mounted with capacitive elements and, by varying the values of capacitive elements, the frequency response can be reconfigured to a desired position. A complete synthesis is presented in order to control the position of center frequency or transmission zero while capacitance values of the capacitive elements and the odd-mode impedance are automatically calculated using the synthesis. To demonstrate the concept, two methods are proposed.

The first method applies RF lumped capacitors as the tuning element. This filter is designed at higher center frequency *f*_*o*_ and, by varying the value of the capacitor, the nominal center frequency can be shifted to a desired position which is lower than the nominal center frequency at *f*_*or*_. The advantage of this design is that the position of reconfigured center frequency can be fixed to a desired position while the value of the capacitors and the odd-mode impedance are calculated automatically using the synthesis. Impedance matching can be achieved by adjusting the impedance values of the ring. The filter is miniaturized up to 71% compared to the ring filter, designed directly at the same reconfigured center frequency, *f*_*or*_.

To further explore the tuning aspect of the reconfigurable filter, varactor-diode with biasing circuit is introduced as the tuning element to electronically tune the center frequency of the ring filter. The advantage of this method is that, even with a small capacitance values, the circuit is capable of tuning continuously to create a frequency-agile characteristic. Finally, both designs are realized on microstrip substrates, simulated, and measured using EM solvers to demonstrate the idea.

## 2. Design of Reconfigurable Ring Filter 

**Figure 1:**
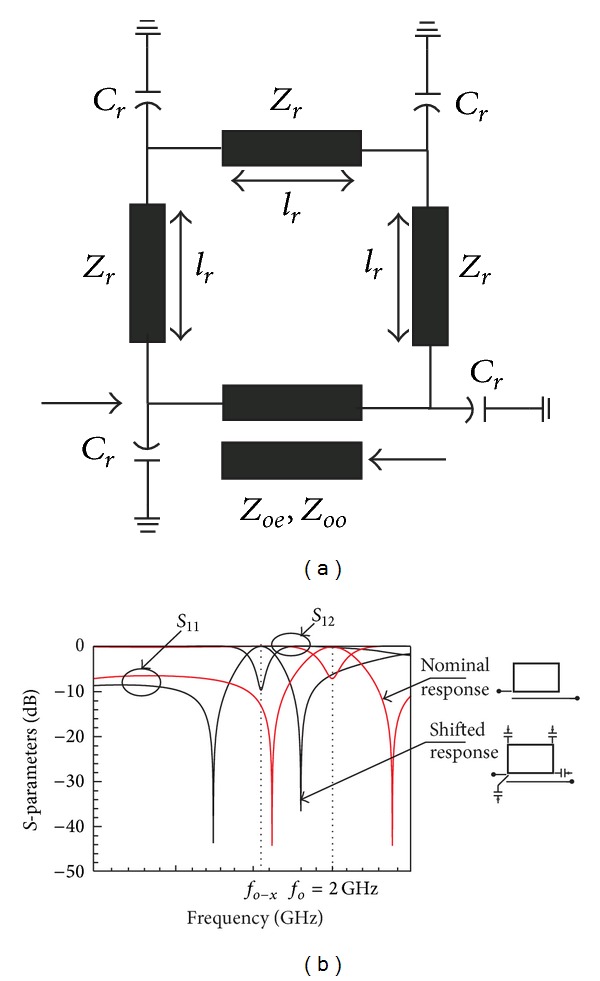
Reconfigurable ring filter electrical length, *l*_*r*_, and four shunted capacitors, designed at center frequency *f*_*o*_ = 2 GHz for *Z*_*r*_ = 85 Ω, *Z*_*oe*_ = 70 Ω, *Z*_*oo*_ = 35 Ω, and *C*_*r*_ = 1 pF. (a) Topology and (b) frequency responses.

A ring resonator is shunted with four capacitive elements, *C*_*r*_, at the edges of the ring lines to vary the nominal electrical length, *l*_*r*_, of the ring. The variation of electrical length depends on the variation values of *C*_*r*_ to reconfigure the nominal center frequency to a new position. As shown in Figure 1(a), the ring is reconfigured with a set of chosen impedances as follows: ring impedance, *Z*_*r*_ = 85 Ω, even-mode impedance, *Z*_*oe*_ = 70 Ω, and odd-mode impedance, *Z*_*oo*_ = 35 Ω, while the tuning element known as reconfigured capacitor, *C*_*r*_, is chosen to be equal to 1 pF.  Figure 1(b) illustrates the frequency responses between the reconfigured ring and the nominal ring without the four reactive elements. As observed, the nominal center frequency, *f*_*o*_, is reconfigured to the left at *f*_*o*-*x*_ due to the changes in electrical length of the ring lines.

### 2.1. Equivalent Circuit and Synthesis

**Figure 2:**
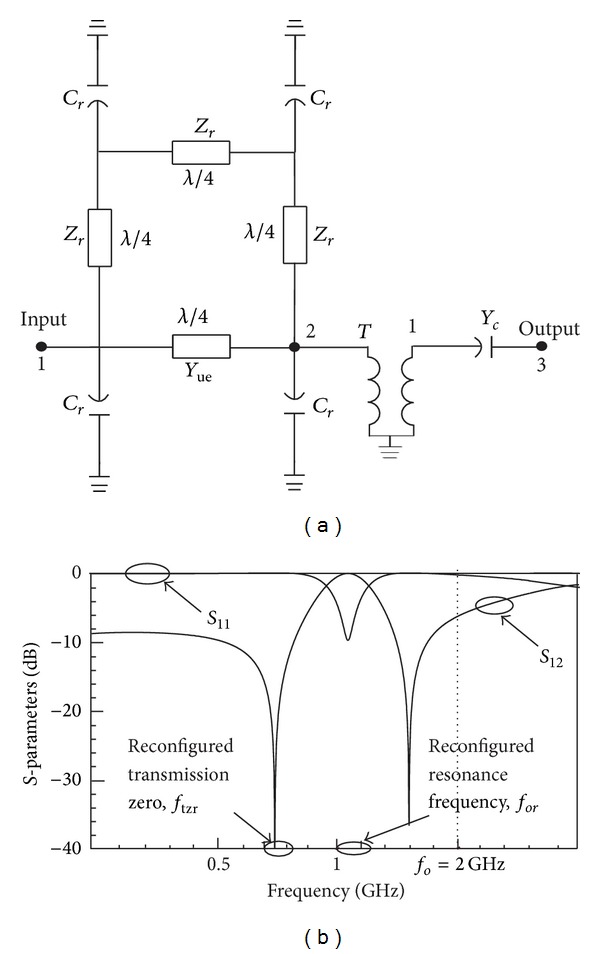
(a) Equivalent circuit diagram of a reconfigurable ring resonator and (b) reconfigured frequency response.

Applying the definitions and parameters of a 3-port coupled-line section given by [12–14], a simplified circuit diagram of the reconfigured ring resonator is constructed as shown in Figure 2(a).

The definitions of transformer *T*, the unit element *Y*_ue_, and the coupling capacitor *Y*_*c*_ are given in (1) to (5) to represent the 3-port coupled-line section while the 3*Z*_*r*_ and reconfigured capacitor *C*_*r*_ represent the ring lines and the tuning elements, respectively. Running a circuit simulation of the diagram in Figure 2(a) with the same value of impedances given earlier in Figure 1(a), where *f*_*o*_ = 2 GHz, *Z*_*r*_ = 85 Ω, *Z*_*oe*_ = 70 Ω, *Z*_*oo*_ = 35 Ω, and *C*_*r*_ = 1 pF, will give the same results as shown in Figure 2(b):

(1) $$\begin{array}{c} Y_{\text{ue}}=Y_{11}-\frac{{Y_{12}}^{2}}{Y_{11}}, \end{array}$$

(2) $$\begin{array}{c} Y_{c}=j\tan\frac{\pi f_{\text{tz}}}{2f_{o}}Y_{11}, \end{array}$$

(3) $$\begin{array}{c} Y_{11}=\frac{\left({1/Z}_{oo}+1/Z_{oe}\right)}{2}, \end{array}$$

(4) $$\begin{array}{c} Y_{12}=\frac{\left({1/Z}_{oo}-1/Z_{oe}\right)}{2}, \end{array}$$

(5) $$\begin{array}{c} T=\frac{Y_{11}}{Y_{12}}. \end{array}$$

**Figure 3:**
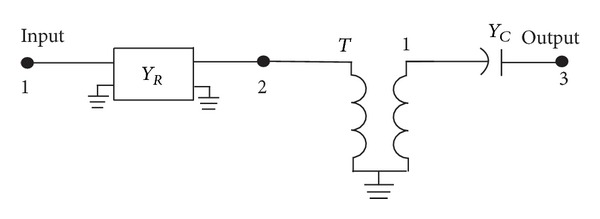
Simplified reconfigured equivalent circuit diagram with a quadripole admittance matrix, *Y*_*R*_.

Next, the reconfigured equivalent circuit in Figure 2(a) is simplified forming a quadripole admittance matrix, *Y*_*R*_, of the closed-loop, while transformer, *T*, and admittance, *Y*_*c*_, at the outer section are as depicted in Figure 3.

At this stage, we need to determine the controlling parameters that influence the position of transmission zeros and the characteristic performance of the nominal ring. Therefore, the quadripole admittance matrix of the closed-loop for the nominal ring is termed as matrix *Y* and can be written as follows:

(6) Y=[Y11Y12Y12Y11],Y11=(4cos⁡⁡(θ)2−3+4YueZrcos⁡(θ)2−YueZr)cos⁡⁡θjZrsin⁡θ(4cos⁡⁡(θ)2−1),Y12=−1+4BYueZrcos⁡⁡(θ)3−BYueZrcos⁡⁡θ(4cos⁡⁡(θ)2−1)jZrsin⁡θ,

with a term given as follows:

(7) $$\begin{array}{c} B=\frac{\left({\tan\left(\theta \right)}^{2}+1\right)}{\sqrt{1+{\tan\left(\theta \right)}^{2}}}. \end{array}$$

Hence, the position of nominal transmission zero, *f*_tz_, can be determined by equating the *Y*_12_ = 0, resulting in

(8) $$\begin{array}{c} 1+4{CY}_{\text{ue}}Z_{r}{\cos\left(\theta \right)}^{3}-{CY}_{\text{ue}}Z_{r}\cos\theta =0. \end{array}$$

Next, the electrical length of the nominal transmission zero at the lower side can be expressed as follows:

(9) $$\begin{array}{c} \theta_{\text{tz}}=\text{arccos}\left(\sqrt{\frac{1-Y_{\text{ue}}Z_{r}}{1+Y_{\text{ue}}Z_{r}}}\right). \end{array}$$

Similarly, the electrical length of the nominal transmission zero can also be represented as

(10) $$\begin{array}{c} \theta_{\text{tz}}=\frac{\pi f_{\text{tz}}}{2f_{o}}. \end{array}$$

Therefore, applying (10) and (11) and for a given nominal transmission zero frequency *f*_tz_, the admittance unit element, *Y*_ue_, can also be written as

(11) $$\begin{array}{c} Y_{\text{ue}}=-\frac{1}{\left(-1+4\cos{\left(\left(1/2\right)\left(\pi f_{\text{tz}}/f_{o}\right)\right)}^{2}\right)Z_{r}}. \end{array}$$

Finally, *Z*_*oo*_ can be written as

(12) $$\begin{array}{c} Z_{oo}=Z_{oe}\left(\frac{2/Z_{oe}-Y_{\text{ue}}}{Y_{\text{ue}}}\right). \end{array}$$

At this stage, the nominal ring can be constructed at arbitrary center frequency, *f*_*o*_, with a chosen set of impedance values of *Z*_*r*_ and *Z*_*oe*_, while *Z*_*oo*_ is calculated using (12). The next step is to synthesize the ring circuit with reconfigured capacitor, *C*_*r*_, shunted at the four edges of the ring line. The purpose of this procedure is to determine the reconfigured frequency response and at the same time calculate the required values of capacitor, *C*_*r*_, using the synthesis.

Next, we need to identify the controlling parameters that influence the shifting of the frequency. By solving the matrix elements of the middle quadripole admittance matrix, *Y*_*R*_, of the reconfigurable ring circuit in Figure 3, one can express in terms of ABCD-matrix the circuit as follows:

(13) $$\begin{array}{c} Y_{R}=\left[\begin{array}{cc} Y_{11r} & Y_{12r}\\ Y_{12r} & Y_{11r} \end{array}\right]. \end{array}$$

Then, by solving the quadripole admittance matrix, *Y*_*R*_, the reconfigured capacitor, *C*_*r*_, can be deduced. This is achieved by equating *Y*_12*r*_ = 0:

(14) Y12r=4Zr2π2ftzr2Cr2Q−1(1−cos⁡⁡(θ)2) −Pcos⁡⁡(θ)+Q−1(4Zr2π2ftzr2Cr2+1) +44Zr2π2ftzr2Cr2(1/2)sin⁡(θ)2cos⁡⁡(θ)2=0.

Finally, by manipulating (14), it leads to the determination of reconfigured capacitor, *C*_*r*_, which can be expressed as follows:

(15) $$\begin{array}{c} C_{r}=-\frac{Q{\cos\left(\theta \right)}^{2}+P\cos\left(\theta \right)-Q-2QZ_{r}\pi f_{\text{tz}r}\cos\left(\theta \right)\sin\left(\theta \right)}{Q{Z_{r}}^{2}\pi^{2}{f_{\text{tz}r}}^{2}\sin{\left(\theta \right)}^{2}}. \end{array}$$

And introducing the terms below to simplify the above equation,

(16) $$\begin{array}{c} P=\sqrt{1+\tan{\left(\theta \right)}^{2}},\\ Q=-\frac{1}{-1+4\cos{\left(\left(1/2\right)\left(\pi f_{\text{tz}}/f_{0}\right)\right)}^{2}}. \end{array}$$

This also means that by fixing the lower side of nominal transmission zero position, *f*_tz_, the impedances can be chosen arbitrarily for a nominal center frequency, *f*_*o*_, by the designer; one can estimate the value of *C*_*r*_ and odd-mode impedance *Z*_*oo*_ which is calculated automatically by (15) and (12), respectively, with respect to the position of reconfigured transmission zero, *f*_tz*r*_.

### 2.2. Application of Synthesis to Control the Position of Transmission Zero

An example of reconfigurable ring filter is designed with a chosen set of impedances given as follows: *Z*_*r*_ = 85 Ω and *Z*_*oe*_ = 70 Ω, and, given by (12), *Z*_*oo*_ is equal to 35 Ω at a nominal center frequency of 2 GHz and transmission zero frequency, *f*_tz_, at 1.6 GHz. In this simulation, the synthesis is applied, and the reconfigured transmission zero, *f*_tz*r*1_, is set at three different positions, which are 1 GHz, 0.9 GHz, and 0.8 GHz. Based on *f*_tz*r*1_, the values of the capacitors, *C*_*r*_, are automatically calculated using (15).

**Figure 4:**
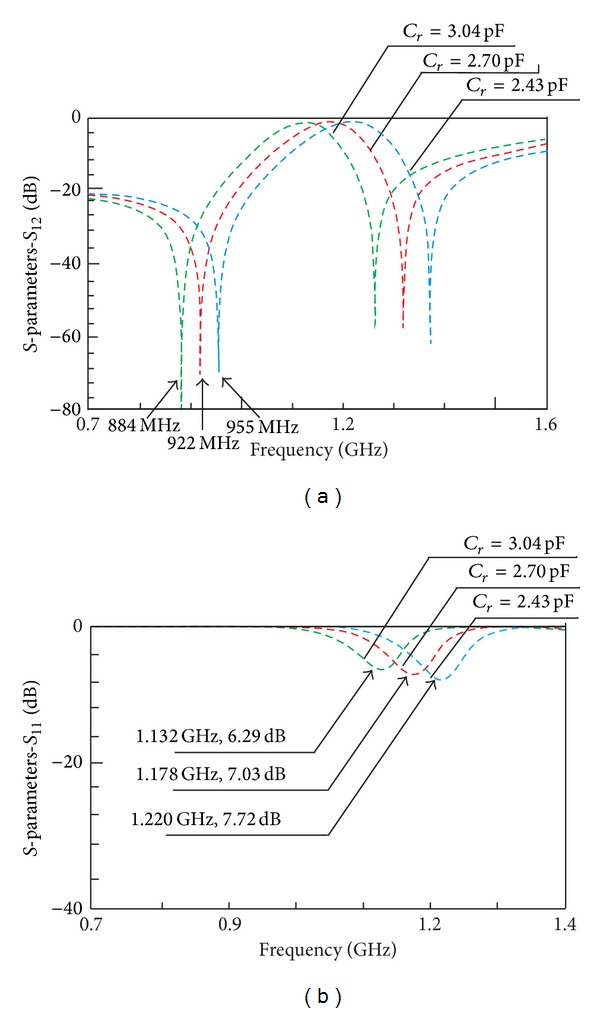
Frequency responses for three different positions of transmission zeros, *f*_tz*r*_, and capacitor, *C*_*r*_, is automatically calculated using the synthesis: (a) *S*_12_ and (b) *S*_11_.

Figures 4(a) and 4(b) depict the frequency responses for three different sets of reconfigured transmission zero. With application of the synthesis, different position of reconfigured transmission zero gives different value of *C*_*r*_. The lower the position of transmission zero is, the higher the *C*_*r*_ value will be.

Table 1 summarized the values of initial setting of reconfigured transmission zero frequency, *f*_tz*r*1_, calculated capacitor, *C*_*r*_, and simulated reconfigured responses of transmission zero frequency, *f*_tz*r*2_, and reconfigured center frequency, *f*_*or*_. It can be observed that the simulated reconfigured transmission zeros, *f*_tz*r*2_, are not at the same position with the initial setting of reconfigured transmission zeros, *f*_tz*r*1_. This is due to the fact that as the frequency shifted to the left the nominal bandwidth is not conserved anymore. Therefore, it is easier and more advantageous to control the reconfigured center frequency than the transmission zeros.

**Figure 5:**
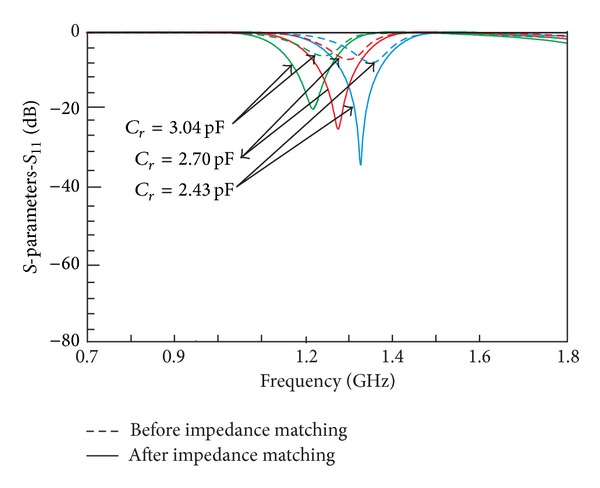
Comparison of frequency responses between initial and after impedances modification for *S*_11_.

It can also be observed that the shifting of frequencies is accompanied by in-band matching problem. Therefore, one needs to be cautious in handling the losses during the implementation stage with some adjustment needed to be done on the impedance values of the ring.  Figure 5 illustrates the performance of return loss before and after the adjustment of impedances, *Z*_*r*_ and *Z*_*oe*_. It can be seen that the return loss has improved exceeding 19 dB when both *Z*_*r*_ and *Z*_*oe*_ are adjusted for impedance matching as compared to the earlier response in Figure 4. However, one has to take note that, with a different set of impedances, the position of center frequency may change accordingly. Finally, return loss and center frequencies before and after adjustments are summarized in Table 2.

### 2.3. Tuning and Application of Synthesis

In a tunable scheme, it is an advantage if one can determine the position of reconfigured center frequency, *f*_*or*_. To achieve this, theory of relative bandwidth (RBW) is applied here in a function of nominal center frequency, *f*_*o*_, and transmission zero, *f*_tz_:

(17) $$\begin{array}{c} \text{RBW}=\frac{\text{BW}}{f_{o}}=\frac{2\left(f_{o}-f_{\text{tz}}\right)}{f_{o}}, \end{array}$$

where BW is bandwidth of the nominal filter.

**Figure 6:**
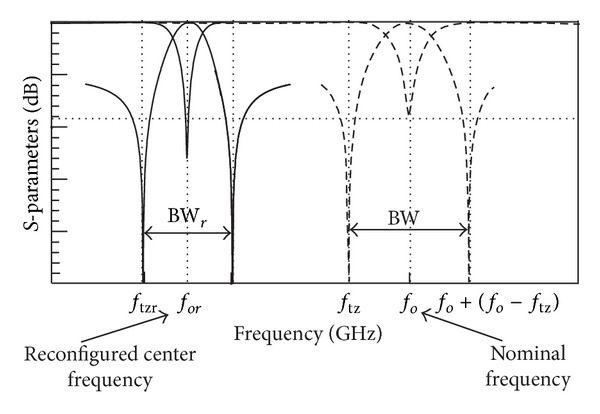
Bandwidth of the nominal and reconfigured filter.

Using relative bandwidth (RBW) concept in (17), relative bandwidth of reconfigured filter, RBW_r_, can be written as follows:

(18) $$\begin{array}{c} {\text{RBW}}_{r}=\frac{{\text{BW}}_{r}}{f_{or}}=2\left(\frac{f_{or}-f_{\text{tz}r}}{f_{or}}\right), \end{array}$$

whereby BW_*r*_ is bandwidth of reconfigured filter with BW > BW_*r*_ as illustrated in Figure 6.

Therefore, to estimate the position of reconfigured center frequency, *f*_*or*_, an assumption has to be made on the reconfigured relative bandwidth, RBW_*r*_. For calculation purpose, let us assume that the relative bandwidth, RBW, is always consistent at any arbitrary center frequency, *f*_*o*_. Therefore, the reconfigured relative bandwidth, RBW_*r*_, can be assumed to be approximately equal to relative bandwidth of nominal filter as follows: RBW_*r*_ ≈ RBW.

By using the expressions in (17) and (18), this can be written as follows:

(19) RBWr≈RBW⟹2fo(fo−ftz)fo≈2for(for−ftzrfor).

Hence, by manipulating (19), the reconfigured center frequency, *f*_*or*_, can be equated as follows:

(20) $$\begin{array}{c} f_{or}\approx \frac{2f_{\text{tz}r}f_{o}}{f_{\text{tz}}}. \end{array}$$

Taking into account the reconfigured relative bandwidth, RBW_*r*_, is only an approximation which is assumed to be equal to the relative bandwidth, RBW. Therefore, to compensate the approximation and obtain a symmetrical response *f*_*or*_ has to be factorized with a tuning parameter of *x*. In other words, (20) can now be written as follows:

(21) $$\begin{array}{c} f_{or}\approx 2\left(\frac{f_{\text{tz}r}f_{o}}{f_{\text{tz}}}\right)x. \end{array}$$

Somehow, to have a good control on filter design, it is practical for the designer to be able to set the position of reconfigured center frequency, *f*_*or*_. Therefore, we introduced a term, *R*_*oc*_, as a ratio of reconfigured center frequency, *f*_*or*_, and nominal center frequency, *f*_*o*_, and this can be expressed as follows:

(22) $$\begin{array}{c} R_{oc}=\frac{f_{or}}{f_{o}}. \end{array}$$

**Figure 7:**
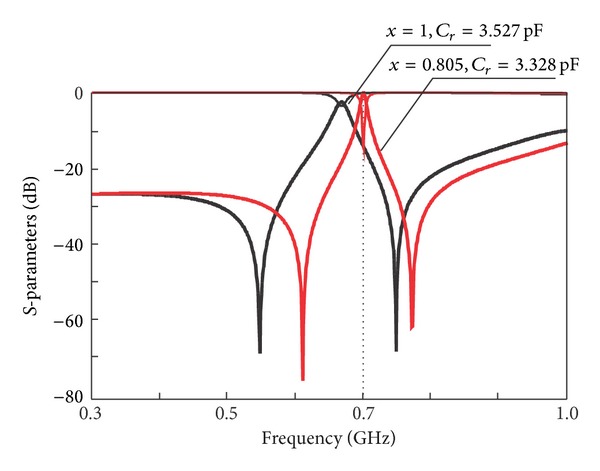
Application of synthesis: frequency responses.

Finally, we can apply the synthesis and predetermine the position of reconfigured center frequency, *f*_*or*_, with initial tuning parameter; *x* is assumed to be 1. Example of application of the synthesis is simulated with a chosen set of impedances given by *Z*_*r*_ = 85 Ω and *Z*_*oe*_ = 70 Ω and given by (12) *Z*_*oo*_ is equal to 35 Ω, designed at center frequency, *f*_*o*_, of 1 GHz. Capacitor, *C*_*r*_, is automatically calculated using (15) to be equal to 3.527 pF. The nominal position of transmission zero, *f*_tz_, is fixed at 0.83 GHz while tuning parameter *x* is tuned accordingly to obtain a symmetrical response. The responses according to variation of *x* are depicted in Figure 7 and summarized in Table 3. It can be seen that, at initial value of tuning parameter *x* = 1, the reconfigured center frequency falls at 668 MHz, while the passband responses exhibit poor matching level. When tuning parameter *x* is tuned to 0.805, the center frequency is reconfigured to 0.7 GHz. To improve the matching level, the impedances are modified as follows: by fixing *Z*_*oe*_, *Z*_*r*_ is modified to 75 Ω while *Z*_*oo*_ is recalculated using (12) and equal to 34.62 Ω while the value of capacitor, *C*_*r*_, is given by (15) to be equal to 3.328 pF.

## 3. **1st** Design: Using Lumped Capacitors

**Figure 8:**
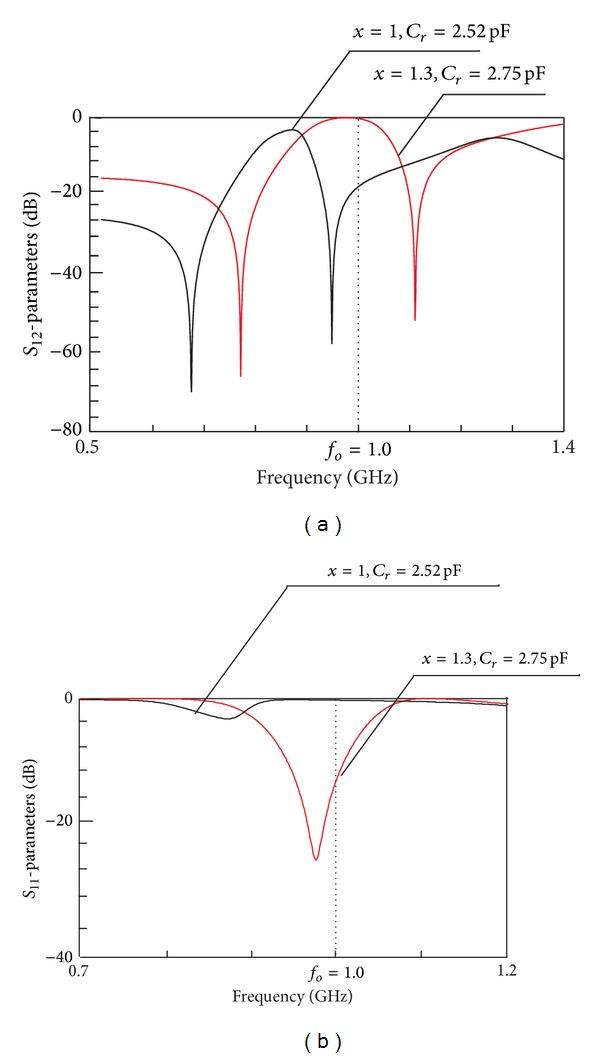
Simulated frequency responses of the reconfigurable ring resonator on ideal circuit using RF lumped capacitors, designed at 2 GHz, *f*_tz_ = 1.6 GHz, with *R*_*oc*_ = 0.5.

For demonstration, a ring filter using four RF lumped capacitors as tuning element to reconfigure its center frequency is proposed. The circuit is designed at *f*_*o*_ = 2 GHz with transmission zero, *f*_tz_, fixed at 1.6 GHz while the reconfigured center frequency, *f*_*or*_, is set at 1 GHz. The impedances of the ring resonator are given with values of *Z*_*r*_ = 85 Ω and *Z*_*oe*_ = 70 Ω and *Z*_*oo*_ given by (12) equals 35 Ω, while capacitor *C*_*r*_ is given by (15) to be equal to 2.52 pF. At initial stage, let us simulate the ideal circuit with parameters set as follows: *x* = 1 and *R*_*oc*_ = 0.5. As shown in Figure 8, the frequency response shifted to the left with lower- and upper-side transmission zeros which are found at 675 MHz and 948 MHz, respectively, while the reconfigured center frequency, *f*_*oc*_, is found at 0.871 GHz with attenuation of 2.93 dB level.

However, this ideal circuit must be tuned to obtain the position of reconfigured center frequency, *f*_*or*_, at 1 GHz. This can be achieved by tuning parameter *x* from 1 to 1.3. For impedance matching, *Z*_*r*_ is modified to 99 Ω, *Z*_*oe*_ is equal to 92 Ω, and *Z*_*oo*_ is automatically calculated using (12) to be equal to 32 Ω while capacitor *C*_*r*_ is given by (15) to be equal to 2.75 pF. As observed in Figure 8, the position of reconfigured center frequency, *f*_*or*_, is shifted to 0.984 GHz with attenuation level improved to be 17.37 dB. The modified lower- and upper-side transmission zeros are found at 780 MHz and 1.103 GHz, respectively.  Table 4 summarized the reconfigured center frequencies and capacitance values according to tuning parameter *x*. The calculated *C*_*r*_ is used as an estimation to choose a suitable value of the capacitor for the implementation of reconfigurable filter on microstrip substrate.

### 3.1. Implementation and Results

**Figure 9:**
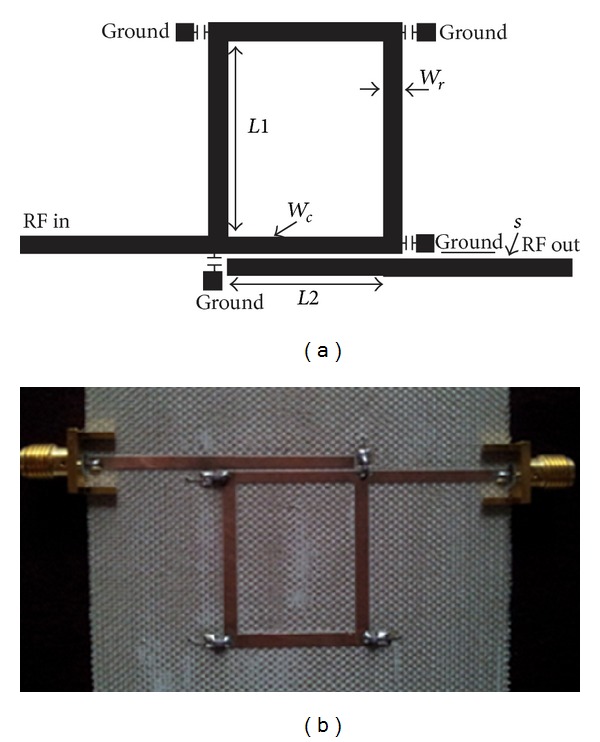
(a) Final layout of the reconfigurable ring bandpass filter; (b) fabricated photo.

During implementation of the filter on microstrip substrate, once again the value of the reconfigured capacitor *C*_*r*_ needs to be adjusted. This is due to the effect of connecting pads (to connect the four lumped capacitors to the ring resonator), via holes, bonding wires, soldering, and the availability of the RF lumped capacitor value available in the market. Based on the calculation of *C*_*r*_ in the previous discussion, RF lumped capacitor with 2.2 pF is chosen. The final layout of the circuit is illustrated in Figure 9(a) with dimensions tabulated in Table 5.

The circuit is then implemented using microstrip technology on substrate Tachonic with characteristics given by *ε*_*r*_ = 4.5, *h* = 1.63 mm, and tan*δ* = 0.0035. As proved by work, a picture of fabricated reconfigurable ring filter is put on view as shown in Figure 9(b) with dimensions tabulated in Table 5.

**Figure 10:**
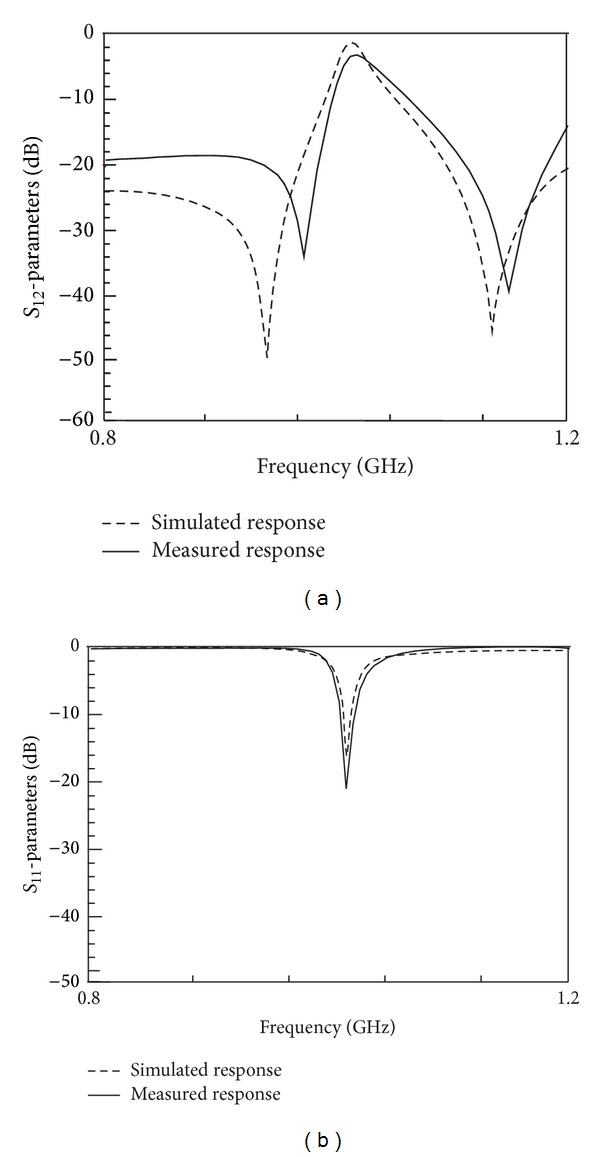
Final responses on microstrip for reconfigurable filter using four capacitors: (a) simulated and measured *S*_11_; (b) simulated and measured *S*_12_.

The simulated and measured frequency responses are depicted in Figures 10(a) and 10(b), respectively. As observed, the simulated reconfigured center frequency attenuates at 0.985 GHz with return loss of 16.72 dB and insertion loss of 2.05 dB. Two transmissions zeros are found at 921 MHz and 1.09 GHz with fractional bandwidth of 1.73%. The measurement results show that the reconfigured center frequency falls at 0.984 GHz and attenuates at 20.78 dB level with insertion loss about 3 dB, while the two transmission zeros are found at 949.4 MHz and 1.104 GHz with fractional bandwidth of 2.03%.

**Figure 11:**
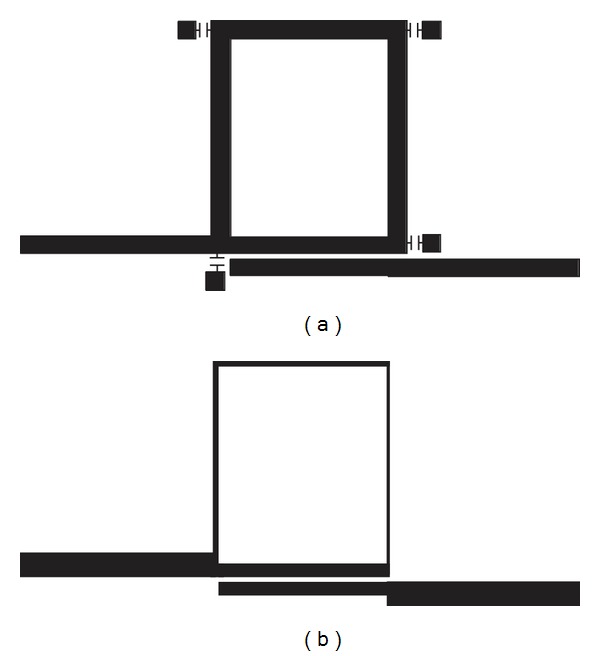
Comparison between filters: (a) reconfigurable ring filter designed at 2 GHz and (b) single mode directly designed at 1 GHz.

Finally, the reconfigurable filter is compared in terms of size with ring filter directly designed at 1 GHz. As illustrated in Figures 11(a) and 11(b), the size of reconfigurable ring filter is greatly reduced and miniaturization has been achieved up to 71% compared to the ring filter directly designed at 1 GHz. The dimensions of the two filters are summarized in Table 6.

## 4. **2nd** Design: Using Varactor-Diodes

The reconfigurable filter of the ring resonator is further explored for tunable filter application using four varactor-diodes to electronically and continuously tune the center frequency. Each varactor-diode is mounted on the microstrip ring resonator circuit via biasing circuit which consists of RF choke resistor, *R*_dc_, and DC block capacitor, *C*_dc_. DC biased voltage is applied to every diode via the resistor *R*_dc_. Thus, the *R*_dc_ has to be large enough to minimize signal leakage. Subsequently, the capacitor, *C*_dc_, has to be sufficient enough to function as DC block to block the DC bias from flowing to the resonator. Finally, every biasing circuit is designed with resistor *R*_dc_ equal to 20 kΩ and capacitor *C*_dc_ equal to 1 nF. The varactors are grounded via hole by drilling the microstrip substrate and the connections are made from varactors to the ground plane using bond wires.

### 4.1. Implementation and Results

For implementation, the varactor-diode model Skyworks SMV1800 is chosen with specifications given as follows: tuning capacitance (*C*_*J*_) = 14.5 pF, package capacitance (*C*_*P*_) = 0.9 pF, bulk resistance (*R*_*S*_) = 2.5 ohm, and package inductance (*L*_*S*_) = 0.8 nH [15]. This model is chosen because of the range of capacitance effect of the varactor which is sufficient to tune the filter from 2 GHz to a desired minimum center frequency of 1 GHz (using (15), *C*_*r*_ is approximately equal to 2 pF).

**Figure 12:**
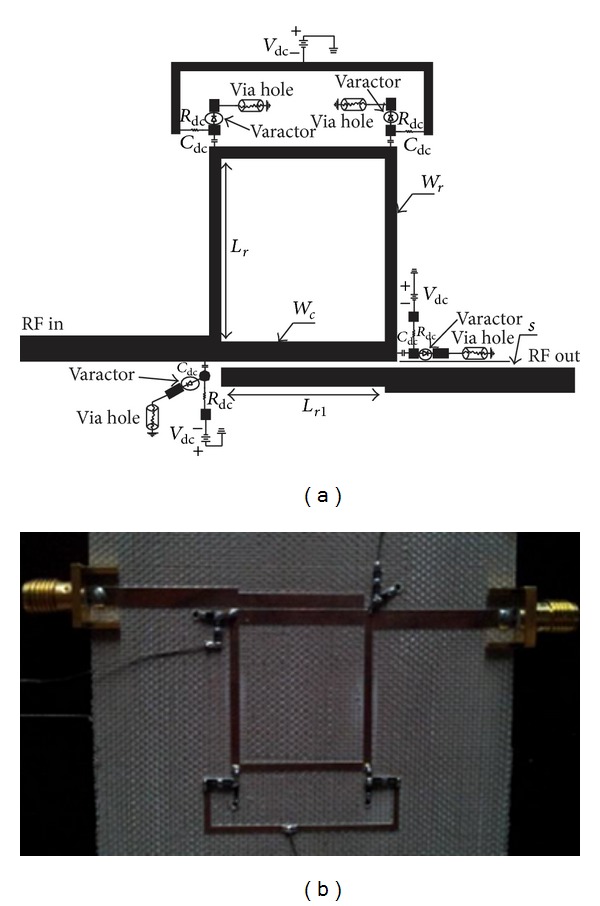
(a) Layout of the electronically reconfigurable ring bandpass filter using four Skyworks SMV 1800 varactors for impedances *Z*_*r*_ = 80 Ω, *Z*_*oe*_ = 75 Ω, and *Z*_*oo*_ = 30 Ω; (b) photo of fabricated filter.

The electronically reconfigurable circuit is designed at 2 GHz with a chosen set of impedances given by *Z*_*r*_ = 80 Ω, *Z*_*oe*_ = 75 Ω, and *Z*_*oo*_ = 30 Ω. The proposed reconfigurable ring bandpass filter is designed using four varactors with biasing circuits which are loaded at the edge of the ring resonator to create capacitance effect to the ring resonator. The circuit is implemented using microstrip technology and substrate Tachonic with characteristics given by *ε*_*r*_ = 4.5, *h* = 1.63 mm, and tan*δ* = 0.0035. The final layout of the filter is depicted in Figure 12(a) with dimensions summarized in Table 7 and a picture of the prototype microstrip reconfigurable bandpass filter is shown in Figure 12(b) as proved by work.

**Figure 13:**
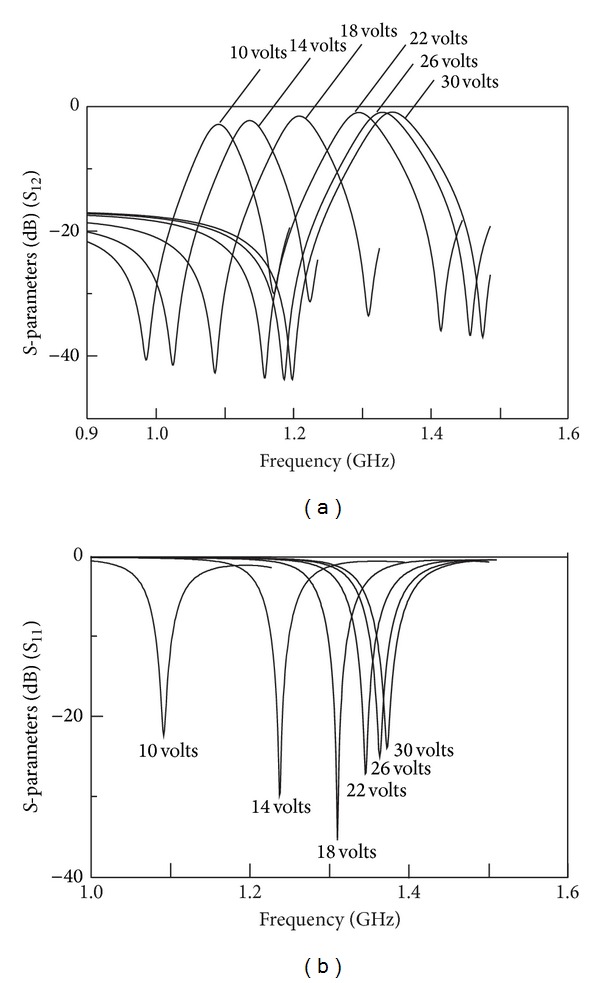
Tuning range from 10 V to 30 V of the electronically bandpass filter for (a) simulated *S*_12_ and (b) simulated *S*_11_.

For a successful reconfigurable filter design, there has to be a trade-off between tunability dynamic range and loss to ensure high filter performance. Therefore, acceptable levels of the frequency responses are chosen in the range from 10 V to 30 V only. From the simulation, it shows that when DC bias voltage is at 10 V, the insertion loss is 2.89 dB and the return loss is 22.49 dB, found at 1.09 GHz. The two transmission zeros exist at 986 MHz and 1.17 GHz, with fractional bandwidth of 4%. When DC bias voltage is at 30 V, the center frequency is found at 1.37 GHz, with insertion loss at 1.05 dB and return loss at 23.98 dB. Two transmission zeros are obtained at 1.22 GHz and 1.51 GHz, respectively, giving a fractional bandwidth of 5%. The simulated frequency responses are illustrated in Figures 13(a) and 13(b).

**Figure 14:**
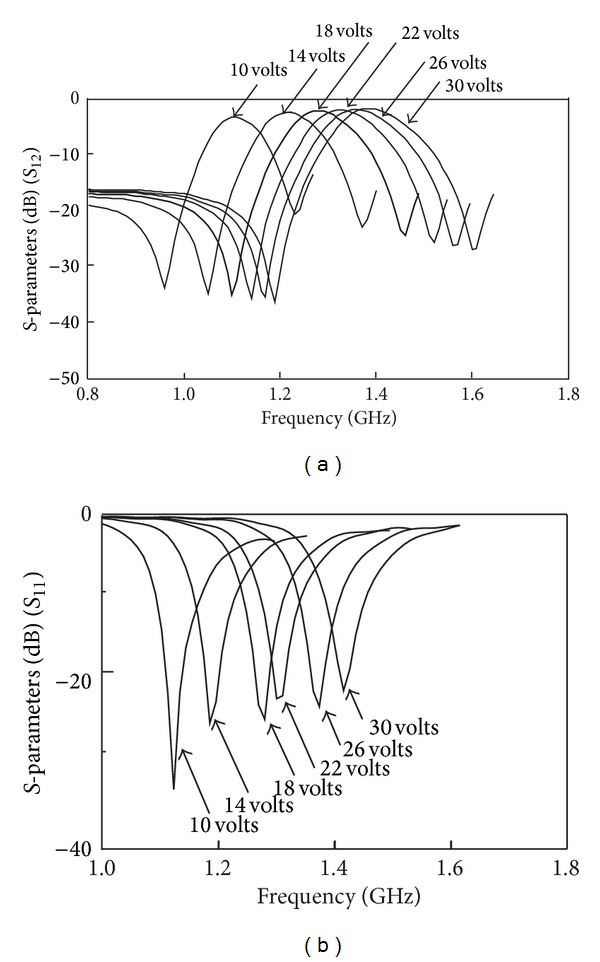
Measured frequency responses for tuning range from 10 V to 30 V: (a) *S*_12_ and (b) *S*_11_.

The measured results are shown in Figures 14(a) and 14(b). The passband characteristics have an insertion loss of 3.12 dB and return loss of 28.96 at 1.10 GHz when the forward bias is at 10 V. Two transmission zeros are obtained at 960 MHz and 1.23 GHz, respectively, with fractional bandwidth of 9%. At forward bias of 30 V, the insertion loss is 1.56 dB while the return loss is 18.62 dB at 1.38 GHz. Two transmission zeros are obtained at 1.19 GHz and 1.60 GHz with fractional bandwidth of 10%.

In general, it is difficult to control both frequency responses and bandwidths along the tuning range. As summarized in Table 8, the measured fractional bandwidth (FBW) is about 10% which is doubled as compared to the simulated FBW in Figure 13. Some possible techniques for better control of bandwidth such as coupling of the nonresonant transmission line of the filter could be employed for future improvement [16]. In terms of tunability, the center frequencies are spreading over 280 MHz which gives 25% achievable tuning range, while the insertion losses from 30 V to 10 V are in the range of 1.56 dB to 3.12 dB, which are within the acceptable specification. As can be observed, there are differences between the simulated and measured insertion losses. This can be attributed to tolerances in the component values and fabrication process.

## 5. Conclusion

This paper explored the use of ring-based resonator topology to develop reconfigurable ring filters. This study had proven that the nominal center frequency of the ring filter can be tuned by introducing capacitive elements which had created variation of electrical length to the ring lines. Synthesis was presented to control the position of reconfigurable center frequency or transmission zero while the value of capacitive elements and the odd-mode impedance are automatically calculated. For demonstration, two reconfigurable filters were proposed using two different tuning techniques. The first prototype made use of four lumped capacitors and the nominal center frequency was successfully reconfigured from 2 GHz to 984.4 MHz, with narrow fractional bandwidth of 2.03%. In terms of size, this filter was successfully reduced by 71% compared to the filter designed directly at 1 GHz. The second prototype made use of hyperabrupt junction varactor-diodes Skywork SMV1800 and the nominal center frequency was tuned in the chosen range of 1.10 GHz to 1.38 GHz spreading over 280 MHz frequency range with achievable tuning range of 25% and fractional bandwidth below 10%. The frequency responses for both filters had shown good passband response, high selectivity with two finite transmission zeros, and narrow bandwidth throughout the tuning range. Finally, both prototypes were simulated and measured to validate the concept.

## Figures and Tables

**Table 1:** Simulated response of a reconfigurable ring resonator designed at center frequency *f*_*o*_ = 2 GHz with the value of impedances given by *Z*_*r*_ = 85 Ω, *Z*_*oe*_ = 70 Ω, and *Z*_*oo*_ = 35 Ω.

Initial setting of reconfigured transmission zero, *f*_tzr1_ (GHz)	Calculated *C*_*r*_ (pF)	Simulated reconfigured responses	
		Transmission zero, *f*_tzr2_ (GHz)	*f*_*or*_ (GHz)
1.00	2.43	0.955	1.220
0.900	2.70	0.922	1.178
0.800	3.04	0.884	1.132

**Table 2:** Summary of values with adjustment impedances *Z*_*r*_ = 80 Ω and *Z*_*oe*_ = 75 Ω while *Z*_*oo*_ is automatically calculated to be equal to 35.066 Ω.

Return loss before adjustment (dB)	Return loss after adjustment (dB)	Position of simulated reconfigured center frequency, *f*_*or*_	
		Before adjustment (GHz)	After adjustment (GHz)
7.72 dB	34.38 dB	1.220	1.202
7.03 dB	25.08 dB	1.178	1.160
6.29 dB	19.97 dB	1.132	1.114

**Table 3:** Summarized values for reconfigured ring designed at nominal center frequency, *f*_*o*_ = 1 GHz, nominal transmission zero, *f*_tz_ = 0.83 GHz, and *R*_*oc*_ = 0.70.

Parameter *x*	1.00	0.805

*Z*_*r*_, *Z*_*oo*_ (Ω)	85.00, 35.00	75.00, 34.62

*Z*_*oe*_ (Ω)	70	70

Reconfigured center frequency, *f*_*or*_ (GHz)	0.668	0.700

Calculated *C*_*r*_ (pF)	3.53	3.328

**Table 4:** Summary of frequency responses on ideal circuit designed at 2 GHz, *R*_*oc*_ = 0.5.

	*x* = 1	*x* = 1.3
Simulated *f*_*or*_	0.871 GHz	0.984 GHz
*Z*_*r*_, *Z*_*oe*_, *Z*_*oo*_ (Ω)	85, 70, 35	99, 92, 32
Calculated *C*_*r*_	2.52 pF	2.75 pF

**Table 5:** Dimensions of the reconfigurable ring filter on Tachonic.

Length *L*_1_ (mm)	Length *L*_2_ (mm)	Ring width *W*_*r*_ (mm)	Coupling width *W*_*c*_ (mm)	Coupling gap *s* (mm)
24.00	18.95	2.27	1.90	0.40

**Table 6:** Dimensions of the two filters on Tachonic.

Ring filter	*f*_*o*_ (MHz)	Insertion loss (dB)	Length (um)	Width (um)	Total dimension (um^2^)
Reconfigured at 1 GHz	984.4	3.00	65.04	30.05	1,954.30
Directly designed at 1 GHz	990.0	1.87	122.94	54.64	6,717.17

**Table 7:** Dimensions of ring filter designed at 2 GHz and biasing components.

Length, *L*_*r*_ (mm)	Length, *L*_*r*1_ (mm)	Coupling width, *W*_*c*_ (mm)	Width ring, *W*_*r*_ (mm)	Gap, *s* (mm)	*R*_dc_ (kΩ)	*C*_dc_ (nF)
22.50	20.20	2.30	1.40	0.24	20.00	1.00

**Table 8:** Measured responses of reconfigurable ring filter, designed at 2 GHz.

DC supply voltage	Poles (GHz)	Transmission zeros (GHz)		Insertion loss (dB)	Return loss (dB)	FBW %
10 volt	1.10	0.96	1.23	3.12	28.96	9
30 volt	1.38	1.19	1.60	1.56	18.62	10
